# The impact of dietary sodium and fructose on renal sodium handling and blood pressure in healthy adults

**DOI:** 10.14814/phy2.70284

**Published:** 2025-03-25

**Authors:** Ronald K. McMillan, Joseph M. Stock, Nathan T. Romberger, Megan M. Wenner, Sheau C. Chai, William B. Farquhar

**Affiliations:** ^1^ Department of Medicine, Division of Clinical Pharmacology Vanderbilt University Medical Center Nashville Tennessee USA; ^2^ Department of Kinesiology and Applied Physiology University of Delaware Newark Delaware USA; ^3^ Department of Kinesiology East Carolina University Greenville North Carolina USA; ^4^ Department of Health Behavior and Nutrition Sciences University of Delaware Newark Delaware USA

**Keywords:** Blood Pressure, Fructose, Renal Function, Salt

## Abstract

Increased dietary sodium is linked to hypertension, but most young adults display “sodium‐resistant” blood pressure (BP), meaning BP is not elevated with sodium loading. In sodium‐resistant rodents, fructose induces salt‐sensitive BP via increased renal sodium reabsorption. Therefore, we tested the impact of fructose and sodium on renal sodium handling and BP in healthy adults, hypothesizing that their combination would impair sodium excretion and increase BP. Thirty‐six participants enrolled in a randomized, double‐blind, crossover trial involving three diets varying in fructose and sodium. On day 7, participants wore ambulatory BP monitors and collected 24‐h urine. Although high sodium increased urinary sodium excretion, excretion was 15% lower with high fructose plus high salt versus high salt alone (235.1 ± 85.0 vs. 277.9 ± 121.2 mmol/24 h, *p* = 0.05). Compared to the recommended diet, high salt alone did not significantly change 24 h. MAP; however, high fructose plus high salt modestly raised 24 h MAP (81 ± 6 vs. 84 ± 7 mmHg, *p* = 0.03). High fructose and high salt increased serum interleukin‐6 concentrations compared to the recommended diet (0.31 ± 0.2 vs. 0.24 ± 0.19 pg/mL, *p* = 0.04). These findings suggest that increased sodium and fructose alter renal sodium handling and BP in young adults.

## 1 INTRODUCTION

The typical Western diet contains excessive sodium and fructose and contributes to the development of hypertension and cardiovascular disease (CVD) (Clemente‐Suárez et al., 2023; Ha, 2014; Hwang et al., 1987; Malik et al., 2016; Soleimani & Alborzi, 2011). There have been multiple studies focusing on the independent effects of sodium and fructose alone on blood pressure (BP), but there is much less work on the potential negative synergistic effects of sodium and fructose on BP in humans (Ha, 2014; Hwang et al., 1987; Wang et al., 2009). One potential effect associated with the combination of fructose and sodium is the development of sodium‐sensitive blood pressure (SSBP). There is a substantial knowledge gap regarding the factors and mechanisms underlying SSBP; yet, there is evidence that suggests the imbalance of renal sodium handling by fructose plays a possible role (Gordish et al., 2017). Since individuals with SSBP have an elevated risk of developing hypertension and increased morbidity and mortality, it is crucial to better understand contributing factors in the development of the SSBP phenotype (He et al., 2021; Morimoto et al., 1997; Weinberger et al., 2001).

Many normotensive adults demonstrate “sodium resistant” BP (i.e., stable BP despite increases in sodium intake) (Weinberger, 1996). However, several preclinical studies demonstrate that increased fructose consumption contributes to SSBP responses in otherwise sodium‐resistant rodent models (Gordish et al., 2017; Hwang et al., 1987; Soleimani & Alborzi, 2011). These studies suggest that a high fructose diet could impair the kidneys' ability to eliminate excess sodium, potentially contributing to elevated BP (Soleimani & Alborzi, 2011; Xu et al., 2017). For example, Sprague Dawley rats, which are considered sodium‐resistant, demonstrated an increase in BP with a 14‐day high‐salt (8% NaCl) chow and moderate fructose (20% in water) intervention (Cabral et al., 2014). These findings are consistent with a similar study in which sodium‐resistant rats were placed on a diet high in both sodium (4% NaCl) and fructose (20%) over 14 days (Gordish et al., 2017). In addition to observing an increase in BP, sodium excretion decreased, suggesting a fructose‐induced increase in sodium reabsorption (Gordish et al., 2017). One possible mechanism for how fructose contributes to the SSBP phenotype is increased angiotensin receptor sensitization, which increases sodium hydrogen exchanger 3 transport within the proximal tubule (Cabral et al., 2014). Additional evidence in rodents suggests fructose augments renal sympathetic nerve activity, leading to an increased renal sodium transport that would not occur during high salt feeding alone (Yokota et al., 2018). Additionally, altered renal sympathetic nerve activity can increase the rate of renin secretion and decrease urinary sodium excretion (Dibona, 2005), which may also contribute to a SSBP response.

Another mechanism that may explain how fructose contributes to the SSBP phenotype is an increase in proinflammatory cytokines. Excess dietary fructose contributes to systemic inflammation and metabolic syndrome by depleting ATP and increasing uric acid production (Hannou et al., 2018; Taskinen et al., 2019). Additionally, sodium loading can contribute to the state of inflammation by increasing the expression of interleukin 6 (IL‐6), an inflammatory cytokine, in blood vessels, tissues, and the gut lining (Tanaka et al., 2014). IL‐6 has also been linked to angiotensin II‐induced salt‐sensitive hypertension (Brands et al., 2010; Hashmat et al., 2016). However, the possible pro‐inflammatory effects of a synergistic diet high in sodium and fructose have not been studied in young healthy adults.

The purpose of this study was to examine the impact of 7 days of high dietary intake of fructose and sodium on renal sodium handling and 24‐h ambulatory BP in young healthy adults, using a double‐blinded randomized crossover approach. We hypothesized that adding high fructose to a high‐sodium diet would impair the kidneys' ability to excrete the excess sodium, increase BP, and increase the inflammatory cytokine IL‐6 in young healthy adults. To test our hypothesis, we assessed changes in urine excretion, blood metabolites, and ambulatory BP.

## 2 MATERIALS AND METHODS

### 2.1 Study design

**FIGURE 1:**
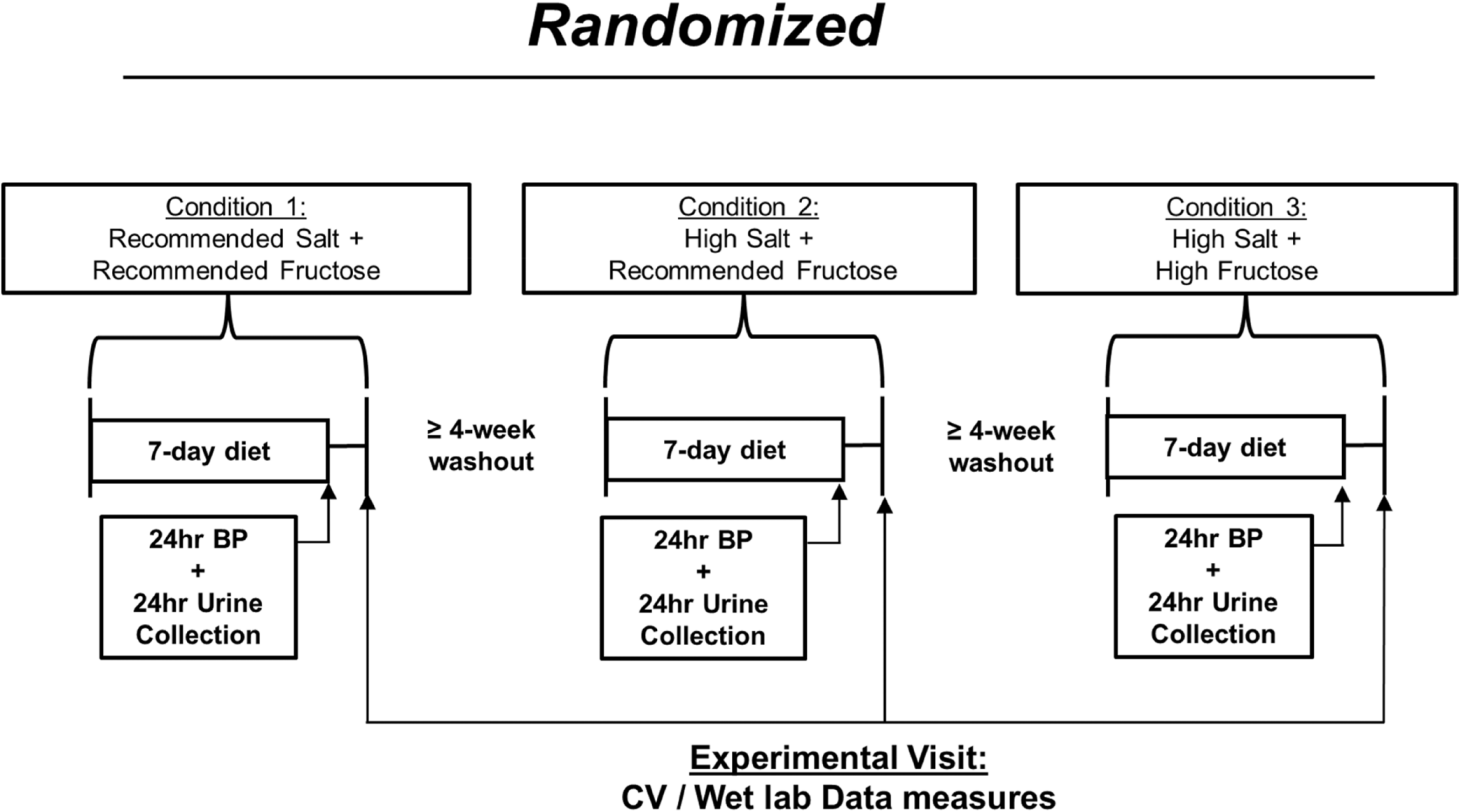
Schematic figure of crossover dietary intervention.

This study utilized a randomized double‐blind crossover design. The study consisted of three 7‐day interventions of a daily low‐fructose drink or a high‐fructose drink in combination with either sodium or placebo pills (described below, in Figure 1). The informed consent and all procedures complied with the Declaration of Helsinki and were approved by the Institutional Review Board at the University of Delaware (IRB #1617405). Participants were recruited from the surrounding area of Newark, DE, by using campus flyers and online advertisements. Informed consent was obtained verbally and in writing before study enrollment. This study is registered on clinicaltrials.gov (identifier: NCT04994418).

### 2.2 Participant screening characteristics

Healthy adults between the ages of 18 and 45 years were enrolled in this study. After obtaining written and verbal consent, participants completed a physical activity readiness questionnaire and a medical history questionnaire. The participants were then assessed for height, weight, and body mass index (BMI). Resting brachial BP measurements were performed in the seated position following ≥5 min of seated rest (Dash 2000, GE Medical Systems). Baseline seated BP measurements were conducted once in both arms. If one arm recorded a higher BP, the arm with the higher BP was used for two additional measurements. The average of the triplicate measures in the higher arm is reported here. Inclusion criteria for this study included resting systolic BP <140 mmHg, resting diastolic BP <90 mmHg, and BMI <30 kg/m^2^ at screening. All participants were free of the following: high BP, high cholesterol, kidney disease, diabetes, thyroid disease, asthma, heart disease, cancer, metabolic, neurological, renal, or pulmonary diseases. Participants were also nonsmokers, not pregnant, and not using antibiotics.

### 2.3 Habitual diet and dietary intervention protocol

Participants received individualized diet counseling by a registered dietitian (RD) based on a 3‐day diet log, which included two weekdays and one weekend day to establish habitual dietary intake. The RD advised participants to limit added sugars and maintain the recommended daily sodium intake (2300 mg). Participants were asked to maintain a similar diet during the three study trials and record their food and water intake during each 7‐day intervention. Each participant was randomly assigned to a crossover dietary intervention, involving a daily fructose drink, either 20 g recommended fructose (RF) or 200 g high fructose (HF), combined with either dextrose (placebo) or salt pills (3900 mg sodium (HS)). The intervention doses were chosen to induce a perturbation based on prior experiments conducted on normotensive adults (Babcock et al., 2020; Perez‐Pozo et al., 2010). Randomization was conducted using research randomizer software (Version 4.0), a widely utilized tool in clinical trials. The study consisted of three 7‐day interventions, separated by at least a 4‐week washout period during which participants resumed their habitual diets. During each 7‐day intervention, participants consumed a daily fructose drink (946 mL) and nine capsules (either sodium or placebo). These were prepared in a metabolic kitchen under the RD's supervision, with the capsules being vegetarian and vegan delayed‐release/acid‐resistant. Fructose powder was ordered from bulk supplements (item #FRUC1KG). Capsules were ordered from Purecaps USA (Size 00 Empty White Vegetarian, item #DJ‐OB84‐JFBQ). Dextrose was provided using NOW FOODS supplier (item #P3973). The salt was provided using Morton's non‐iodized table salt. The Capsules were filled using a capsule loading machine, size 00 manufactured by All‐In Capsule.

To minimize gastrointestinal discomfort from excessive salt and fructose consumption, participants ingested three capsules with whole foods at different times each day, ensuring at least a 6‐hr interval between doses. They consumed the fructose drink in moderation, with at least a 2‐h interval between each 68 mL serving. Participants logged any additional water consumption and any unconsumed supplements. On the final day of each intervention, participants were instructed to avoid caffeine, alcohol, and exercise for at least 24 h before lab testing.

### 2.4 Physical activity monitoring

Participants wore an accelerometer (ActiGraph wGT3X‐BT, Pensacola, FL) on their dominant side hip during each 7‐day dietary intervention, excluding periods of sleep and activities with water such as swimming or showers. Participants logged the periods during which the accelerometer was worn to enhance adherence and the accuracy of the data. Data was considered valid if there were a minimum of 3 days with a wear time of ≥8 h/day (Evenson & Wen, 2015). The accelerometer data was evaluated using ActiLife software for physical activity, step count, and sedentary time.

### 2.5 24‐h ambulatory blood pressure and urine collection

On the last day of each diet, the participants were asked to wear a BP monitor (Oscar 2, Suntech) on their nondominant upper arm for 24 h. This BP cuff was set to automatically take the participants' BP every 20 min during awake hours and every 30 min during sleeping hours. The monitor records and saves each BP measurement automatically. The participants documented their sleep and wake times. The BP data collected during periods of sleep and wakefulness were used to examine nocturnal BP dipping (Babcock et al., 2020; Brian et al., 2017; Greaney et al., 2012; Matthews et al., 2015). Nocturnal dipping was calculated as the percent decline in nocturnal BP: nocturnal dipping % = ((average daytime BP−average nocturnal BP)/average daytime BP) × 100. Observations were omitted if (1) quality was poor or (2) fewer than 20 readings were recorded during the daytime and/or fewer than seven readings were recorded at night (O'Brien et al., 2013). During the same 24 h that the participant wore the BP monitor on their arm, participants also collected urine for 24 h. The urine sample was analyzed to assess urinary sodium excretion, osmolality, urine specific gravity, and creatinine clearance.

### 2.6 Experimental visit

Upon arrival at the laboratory, each participant provided a spot urine sample. Body weight was recorded at the beginning of each study visit (Tanita Body Composition Analyzer, Model TBF‐300A; Arlington Heights, IL). Participants then laid in the supine position for 15 min before a venous blood sample was collected.

### 2.7 Blood and urine analysis

Venous blood samples were analyzed for serum electrolyte concentrations (EasyElectrolyte Analyzer; Medica, Beford, MA, USA) and plasma osmolality (3D3 Osmometer; Advanced Instruments, Norwood, MA). Venous blood samples were also analyzed for hemoglobin (Hb 201+; Hemocue, Lake Forest, CA, USA) and hematocrit (Pre‐calibrated Clay Adams, Readacrit Centrifuge; Becton Dickinson, Sparks, MD, USA). Additionally, urine‐specific gravity was determined for the spot and 24‐h urine samples. Female participants' spot urine samples were used to confirm pregnancy status (hCG cassettes, Moore Medical).

Participants collected their urine for 24 h on the last day of each diet, and the overall volume of the urine was measured. Testing was conducted to measure urine osmolality (3D3 Osmometer; Advanced Instruments, Norwood, MA) and sodium (Na^+^), potassium (K^+^) and chloride (Cl^−^) concentrations in the urine (EasyElectrolyte Analyzer; Medica, Beford, MA, USA). Osmometer reagents used: Advanced Instruments Calibration Standards used for Osmometer: Part number: 3LA011 Description: 100 mOsm/kg H_2_O Calibration Standard, Part number: 3LA151D, Description: 1500 mOsm/kg H_2_O Calibration Standard, Part number 3LA029 Description: 290 mOsm/kg H_2_O Calibration Standard. Electrolyte analyzer reagents used: Fisher Scientific Catalog No. 22‐515‐443, Description: Medica EasyElectrolytes Analyzer: Diluents. Part number: 6204, Description: Medica EasyElectrolytes Reference Sensor. A sample of the 24‐h urine and a serum sample were provided to LabCorp for the measurement of creatinine clearance. Additional calculations included sodium excretion, free water clearance, and osmolar clearance using standard formulas.

### 2.8 Calculation of 24‐h sodium excretion, free water clearance, and osmolar clearance

The calculation of 24‐h urinary sodium excretion was based on 24‐h urine volume and the measurement of sodium concentration in a 24‐h urine sample. The equation is provided below:

$$\begin{array}{cc} 24\;h\;\text{urinary}\;\text{sodium}\;\text{excretion}=\; & \mathrm{Na}\;\text{concentration}\;\left(\text{mmol}/L\right)\\ & \times \text{volume}\;\left(L/24\;h\right) \end{array}$$

The calculation of 24‐h free water clearance is provided below:

$$\begin{array}{c} 24\;h\;\text{free}\;\text{water}\;\text{clearance}=\text{volume}\;\left(\mathrm{mL}/\min\right)\\ \times \left[1-\left(\text{urine}\;\text{osmolality}/\text{plasma}\;\text{osmolality}\right)\right] \end{array}$$

The calculation of 24‐h osmolar clearance is provided below:

$$\begin{array}{c} 24\;\text{h}\;\text{osmolar}\;\text{clearance}=\text{urine}\;\text{osmolality}\\ \times \left(\text{volume}\;\left(\mathrm{mL}/\min\right)/\text{plasma}\;\text{osmolality}\right) \end{array}$$

### 2.9 Interleukin‐6 concentrations in serum

Serum IL6 was measured by Simoa technology on a SRX analyzer, according to the manufacturer's instructions (Quanterix, Billerica, MA). The Simoa IL6 kit (Quanterix) was used. Briefly, serum samples were thawed at 22°C, gently mixed, and centrifuged at 2000 RPM for 5 min at 22°C. Samples were diluted 1:4 with sample diluent and bound to paramagnetic beads coated with a capture antibody specific for human IL6. Antibody‐coated beads were incubated with a biotinylated anti‐IL6 detection antibody, which in turn were labeled with a streptavidin‐galactosidase complex.

### 2.10 Statistical analysis

We conducted an a priori power analysis based on an effect size of 0.25 using previously collected data to determine the appropriate sample size (GPower 3.1) (Matthews et al., 2015). Assuming a moderate effect size, it was determined that a total of 35 participants would be needed to provide at least 90% power with α set at 0.05 to detect a 5‐mmHg difference in MAP when comparing the three dietary conditions. An increase in BP of 5 mmHg has been associated with an increased risk for CV diseases (Maaliki et al., 2022). Repeated measures (RM) one‐way analysis of variance (ANOVA) models were used to assess differences in BP and renal parameters (e.g., sodium excretion and free water clearance) across all three conditions. For characteristics that demonstrated a significant difference (*p* < 0.05) among the experimental measures, we performed post hoc comparisons using Tukey's multiple comparison. Data is reported as mean ± SD with the α level set a priori at 0.05. Exploratory sex comparisons were conducted using 2 × 3 (sex × condition) RM ANOVAs. Statistics were performed using GraphPad Prism 8 (GraphPad Software Inc., La Jolla, Calif., USA).

## 3 RESULTS

### 3.1 Baseline participant characteristics

Thirty‐six participants completed this study. Participant screening characteristics are presented in Table 1. Participants were young (18–45 yr), healthy, and nonobese. Participants consumed an average of 2055 ± 698 Calories in their habitual diet prior to starting the dietary intervention. The participants were counseled by an RD to limit their daily intake of sodium to 2300 mg and eliminate excess added sugars outside of the supplements provided by the study.

**TABLE 1:** Screening characteristics.

*N*, men/women	36 (17/19)
Race, White/Black/Asian	27/2/7
Age, years	30 ± 8
Weight, kg	70.5 ± 13.3
Height, cm	173.3 ± 10.3
BMI, kg/cm^2^	23.1 ± 3.7
Systolic BP, mmHg	113 ± 12
Diastolic BP, mmHg	67 ± 8
MAP, mmHg	80 ± 16

*Note*: Data are expressed as mean ±SD.

Abbreviations: BMI, body mass index; BP, blood pressure; MAP, mean arterial pressure; *N*, number of participants.

### 3.2 Renal measures

**FIGURE 2:**
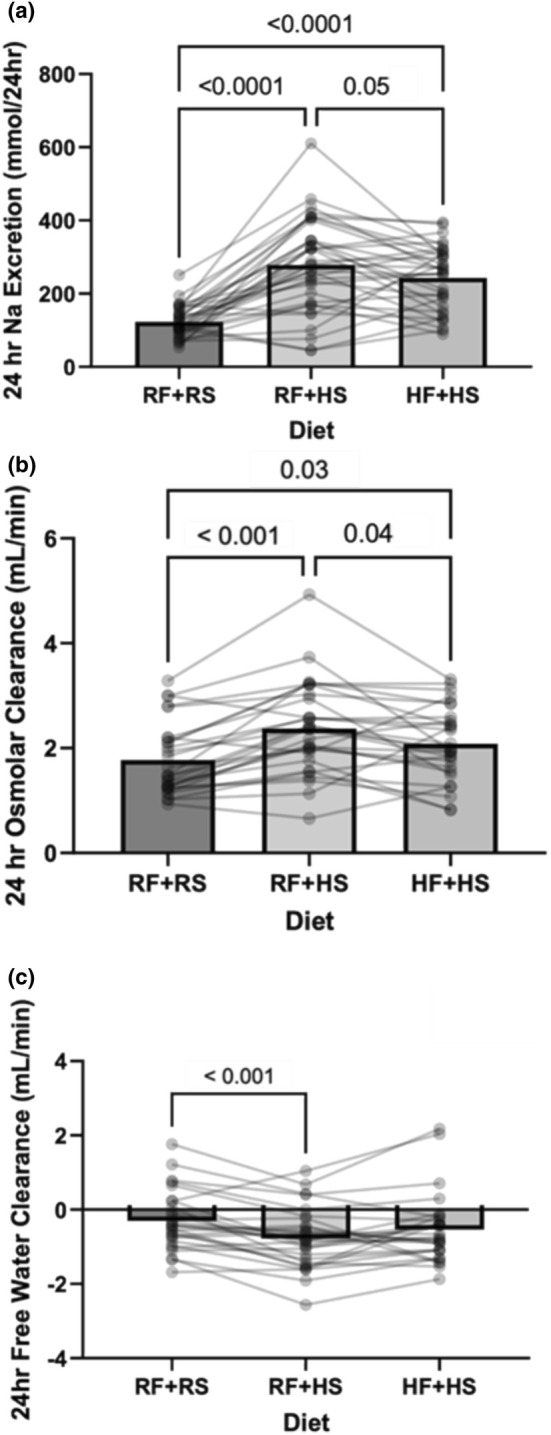
Renal perturbation measures following each dietary intervention of recommended fructose plus recommended salt (RF + RS), recommended fructose plus high salt (RF + HS), and high fructose plus high salt (HF + HS). (a) 24‐h urinary sodium excretion *N* = 35. RM one‐way ANOVA: *p* < 0.0001. (b). 24‐h free osmolar clearance results following each dietary intervention *N* = 28. RM one‐way ANOVA: *p* < 0.0001. (c). 24‐h free water clearance results following each dietary intervention. *N* = 28. RM one‐way ANOVA: *p* = 0.001. Data are presented as individual and mean values. Significant differences were followed with post hoc pairwise comparisons using Tukey's multiple comparison.

As depicted in Figure 2, sodium excretion was significantly higher in both sodium loading conditions compared to the recommended sodium condition (Figure 2a, *p* < 0.001). The addition of high fructose blunted sodium excretion by 15% compared to the RF + HS condition (HF + HS: 235.1 ± 85.0 mmol/24 h vs. RF + HS: 277.9 ± 121.2 mmol/24 h, *p* = 0.05). Urine osmolality increased in both RF + HS and HF + HS conditions when compared to RF + RS (Table 2, *p* = 0.01). Osmolar clearance increased in both RF + HS and HF + HS when compared to the RF + RS condition (RF + HS: 2.36 ± 0.9 mL/min, HF + HS: 2.08 ± 0.7 mL/min vs. RF + RS: 1.79 ± 0.7, *p* < 0.001; Figure 2b). Free water clearance decreased in the RF + HS condition when compared to the RF + RS condition (RF + HS: −0.7 ± 0.8 mL/min vs. RF + RS: −0.33 ± 0.76 mL/min, *p* < 0.001), but there was not a significant difference in free water clearance between the RF + RS and HF + HS conditions (RF + RS: −0.33 ± 0.76 mL/min vs. HF + HS: −0.54 ± 0.9 mL/min, *p* = 0.11; Figure 2c). Urine flow rate remained unchanged in response to increased sodium or the combination of sodium and fructose (RM one‐way ANOVA *p* = 0.10). There was no impact on creatinine clearance due to the diet (Table 2, *p* = 0.15). The intervention did not induce any changes in hydration status based on the 24 h urine specific gravity (Table 2, *p* = 0.30).

**TABLE 2:** Laboratory and urinary measures after each intervention.

	RF + RS	RF + HS	HF + HS	*p*	*N*
Heart rate, (bpm)	73 ± 10	72 ± 11	71 ± 10	0.71	33
Weight, (kg)	68 ± 13	66 ± 13	68 ± 13	0.40	36
Serum sodium, (mmol/L)	138.2 ± 1.5	138.2 ± 1.9	139.2 ± 2.8	0.23	26
Serum potassium, (mmol/L)	4.02 ± 0.34	3.95 ± 0.26	4.64 ± 0.40	0.58	26
Serum chloride, (mmol/L)	105.1 ± 1.7	105.9 ± 1.7	105.4 ± 1.5	0.13	26
Plasma osmolality, (mOsm/kg H_2_0)	287.0 ± 6.6	288.3 ± 5.4	288.0 ± 3.4	0.37	28
Hemoglobin (g/dL)	13.4 ± 1.7	13.2 ± 1.6	13.2 ± 1.9	0.17	32
Hematocrit, (%)	42.3 ± 3.5	41.6 ± 3.4	42.4 ± 3.5	0.13	32
Glucose (mg/dL)	88.5 ± 8.8	87.9 ± 6.3	90.6 ± 7.1	0.11	26
Total cholesterol (mg/dL)	166.0 ± 30.1	164.6 ± 26.5	168.3 ± 34.0	0.43	26
HDL, cholesterol, (mg/dL)	53.8 ± 13.6	53.0 ± 14.6	50.7 ± 13.1	0.10	26
VLDL, cholesterol, (mg/dL)	14.5 ± 4.5	16.5 ± 8.0	21.7 ± 15.8*	**0.02**	26
LDL, cholesterol, (mg/dL)	97.7 ± 25.8	95.0 ± 21.5	96.0 ± 24.2	0.65	26
Triglycerides, (mg/dL)	75.1 ± 28.9	88.4 ± 50.0	117.2 ± 88.5*	**0.01**	26
Uric acid, (mg/dL)	4.7 ± 1.2	4.2 ± 1.2*	4.2 ± 1.2*	**<0.01**	26
Serum creatinine, (mg/dL)	0.87 ± 0.2	0.82 ± 0.2	0.83 ± 0.2	0.07	21
Urinary sodium excretion, (mmol/24 h)	121.5 ± 42.2	277.9 ± 121.2*	235.1 ± 85.0*, ^†^	**<0.01**	34
24‐h urine osmolality, (mOsm/kg H_2_0)	421.4 ± 219.3	539.8 ± 253.4*	517.1 ± 243.0*	**0.01**	34
Urine flow rate (mL/min)	1.55 ± 0.9	1.75 ± 1.4	1.54 ± 1.0	0.10	36
24‐h urine specific gravity	1.011 ± 0.005	1.012 ± 0.006	1.012 ± 0.006	0.30	36
Free water clearance (mL/min)	−0.33 ± 0.8	−0.80 ± 0.8*	−0.54 ± 0.9	**<0.01**	28
Osmolar clearance (mL/min)	1.79 ± 0.7	2.36 ± 0.9*	2.08 ± 0.7*, ^†^	**<0.01**	28
Creatinine clearance (mL/min)	109.5 ± 25.2	118.4 ± 32.1	110.4 ± 29.1	0.15	19

*Note*: Recommended fructose plus recommended salt (RF + RS), recommended fructose plus high salt (RF + HS), and high fructose plus high salt (HF + HS). Data are expressed as mean ± SD. Significant differences were followed with post hoc pairwise comparisons using Tukey's multiple comparison.

Abbreviation: *N*, number of participants.

* Denotes *p* < 0.05 for respective condition versus RF + RS.

^†^ Denotes *p* < 0.05 for the respective condition versus RF + HS. Boldface indicates significant values.

### 3.3 24‐h ambulatory BP monitoring

**FIGURE 3:**
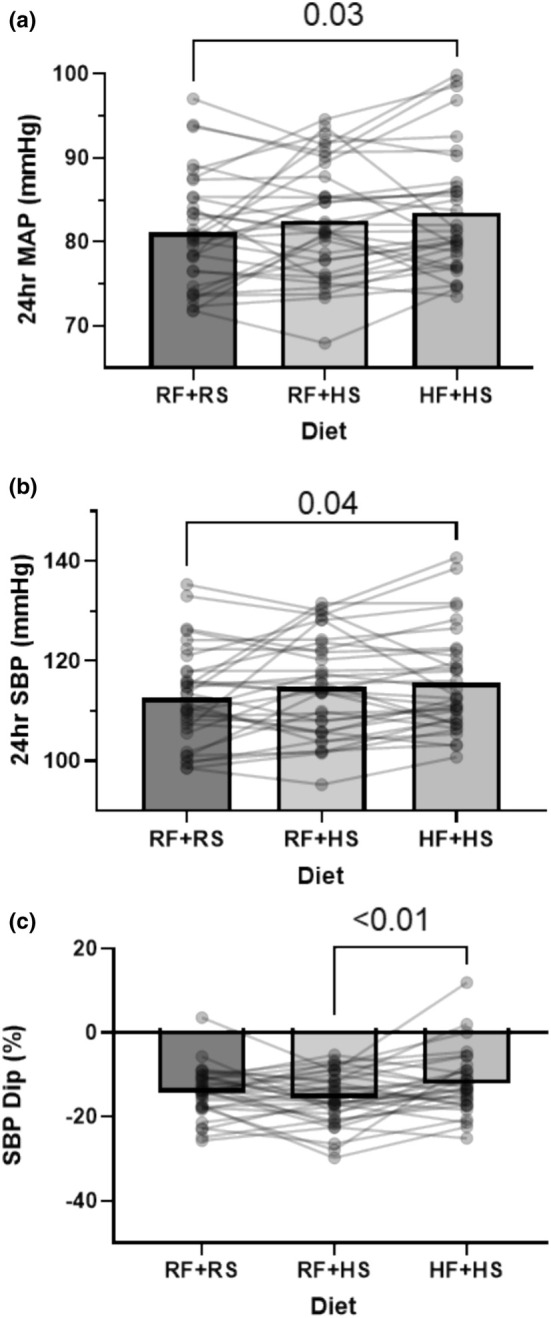
24‐h ambulatory BP results following each dietary intervention of recommended fructose plus recommended salt (RF + RS), recommended fructose plus high salt (RF + HS), and high fructose plus high salt (HF + HS). (a) Mean Arterial Pressure. *N* = 33. RM one‐way ANOVA: *p* = 0.03. (b) Systolic blood pressure. *N* = 33 RM one‐way ANOVA: *p* = 0.04 (c). Nocturnal blood pressure dip. *N* = 33. RM one‐way ANOVA *p* = 0.009. Data are presented as individual and mean values. Significant differences were followed with post hoc pairwise comparisons using Tukey's multiple comparison.

**FIGURE 4:**
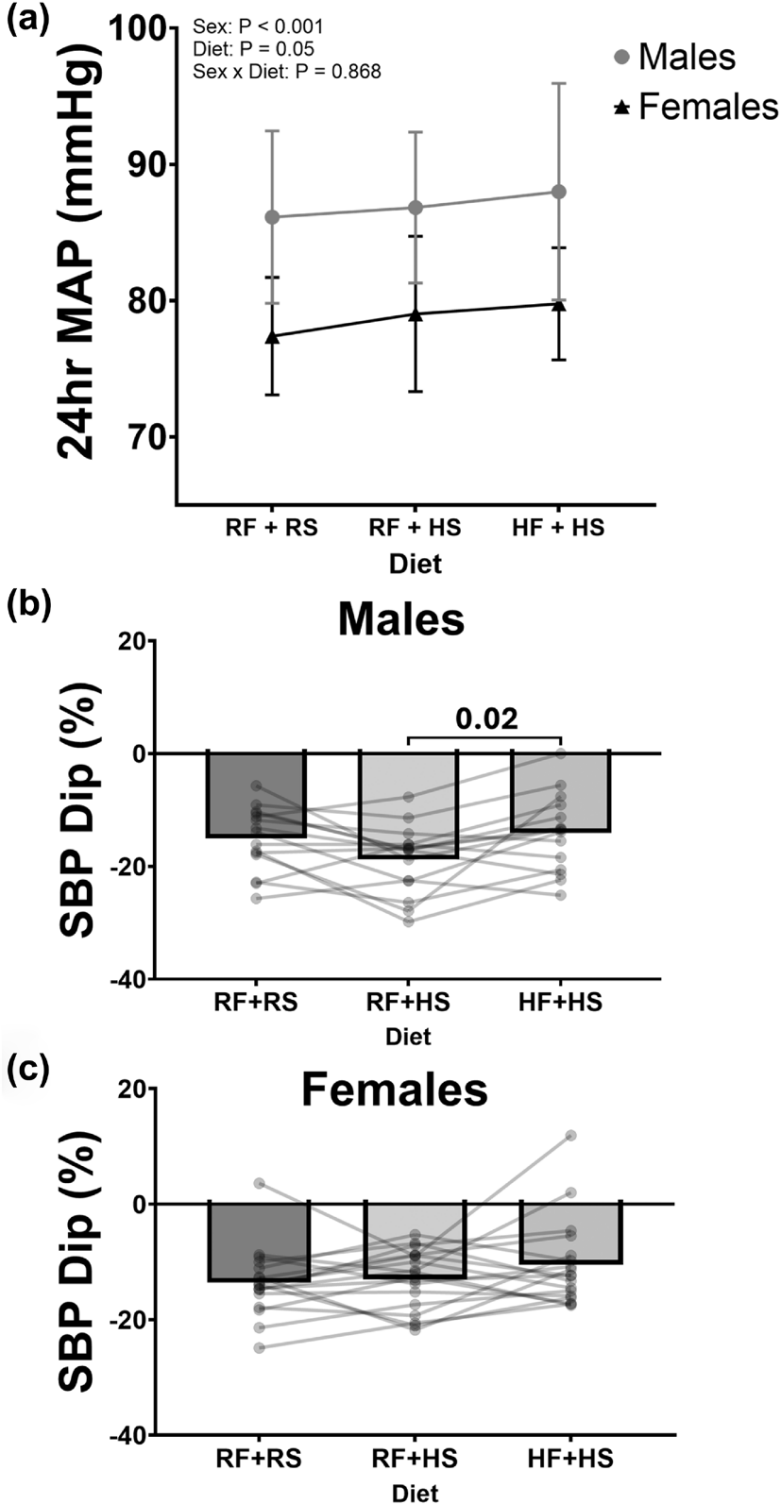
Sex comparison of 24‐h ambulatory BP results following each dietary intervention of recommended fructose plus recommended salt (RF + RS), recommended fructose plus high salt (RF + HS), and high fructose plus high salt (HF + HS). (a) Mean arterial pressure. Males, *N* = 15, Females, *N* = 18. Two‐way ANOVA: Sex *p* = <0.001, Diet *p* = 0.04, Sex × Diet *p* = 0.90. (b) Nocturnal blood pressure dip. Males, *N* = 15 RM one‐way ANOVA: *p* = 0.02. (c) Nocturnal blood pressure dip. Females, *N* = 18, RM one‐way ANOVA: *p* = 0.12. Data are presented as mean values and SD for panel (a). Data are presented as individual and mean values for panels (b) and (c). Significant differences were followed with post hoc pairwise comparisons using Tukey's multiple comparison.

Participants wore an ambulatory BP monitor for 24 h. Participant data were excluded due to improperly wearing the BP cuff or being noncompliant with the protocol (*N* = 3). 24‐h MAP modestly increased during the HF + HS condition when compared to the RF + RS condition (Figure 3a, HF + HS: 84 ± 7 vs. RF + RS: 81 ± 6 mmHg, *p* = 0.03). A similar effect was observed for SBP (*p* = 0.04; Figure 3b) and DBP (*p* = 0.053; data not shown). Figure 3c presents the effect the diet had on nocturnal dipping. Systolic BP dipping remained above the clinically relevant 10% threshold in all three conditions. Although there were no significant differences between the RF + RS and HF + HS conditions, the HF + HS condition exhibited statistically less systolic BP dipping compared to the RF + HS condition (Figure 3b, HF + HS: −12 ± 7% vs. RF + HS: −16 ± 6, *p* = 0.007). Figure 4 demonstrates exploratory comparisons of the impact of sex on the intervention. There was a significant effect of the diet on BP (Figure 4a, *p* = 0.05). Males showed an overall higher BP than females (Figure 4a, *p* < 0.001); however, there were no significant differences in how the diet influenced BP in each sex (Figure 4a, Sex × Diet, Interaction, *p* = 0.90). We present a sex comparison of nocturnal SBP dipping, showing that although SBP dipping was significantly lower in HF + HS compared to RF + HS in males (Figure 4b, males RF + HS: −19 ± 6% vs. HF + HS: −14 ± 7%, *p* = 0.02) there were no significant differences in SBP dipping between the diets in females (Figure 4c, females RF + HS: −13 ± 5% vs. HF + HS: −11 ± 7%, *p* = 0.11).

### 3.4 IL‐6 in serum

**FIGURE 5:**
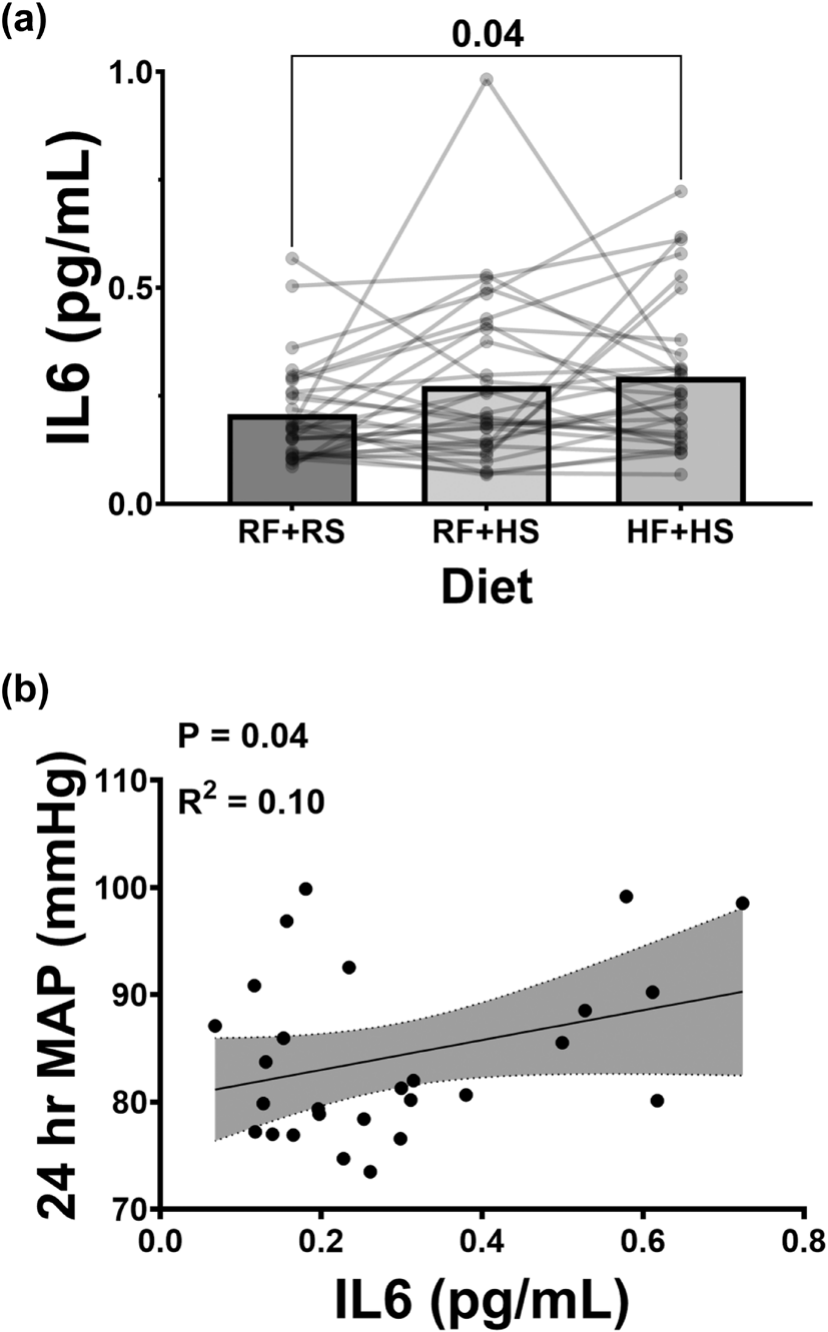
Assessment of IL6 in serum and the BP correlations. (a) Interleukin 6 levels detected in serum following each dietary intervention of recommended fructose plus recommended salt (RF + RS), recommended fructose plus high salt (RF + HS), and high fructose plus high salt (HF + HS). *N* = 30, RM one‐way ANOVA: *p* = 0.04. Data are represented as individual and mean values. Significant differences were followed with post hoc pairwise comparisons using Tukey's multiple comparison. (b) Correlations between each dietary intervention of recommended fructose plus recommended salt (RF + RS), recommended fructose plus high salt (RF + HS), and high fructose plus high salt (HF + HS) and the respective BP measures were analyzed using linear regression and significance was computed with a one‐tailed Pearson test.

Serum was collected from each participant at the end of each diet to assess IL‐6. Figure 5 presents the levels of interleukin‐6 (IL‐6) detected in serum following each diet. The serum IL‐6 concentration increased modestly after the HF + HS condition compared to the RF + RS condition (Figure 5a, 0.31 ± 0.2 vs. 0.24 ± 0.19 pg/mL, *p* = 0.04). There was no significant difference when comparing the RF + RS condition to the RF + HS condition (*p* = 0.17). We also found a modest correlation between IL‐6 and 24‐h MAP (Figure 5b, *r* ^2^ = 0.10, *p* = 0.04) during the HF + HS diet. There was no significant correlation during the other two diets.

### 3.5 Experimental lab measures

Participants self‐reported water intake, capsules remaining, and fructose beverage remaining at the end of each diet. There were no statistically significant differences in additional water intake (RF + RS: 1508 ± 828, RF + HS: 1715 ± 858, and HF + HS: 1715 ± 1005 mL/day, *p* = 0.31), capsules remaining (RF + RS: 0.11 ± 0.4, RF + HS: 0.11 ± 0.4, and HF + HS: 0.11 ± 0.4 capsules/day, *p* = 0.99), or fructose beverage remaining after each diet (RF + RS: 27 ± 74, RF + HS: 43 ± 77, and HF + HS: 68 ± 95 mL/day, *p* = 0.09). Physical activity was not different between any of the three diets (*p* > 0.05, Table 3). Lab and urinary measures are presented in Table 2. Body weight was not different between the diets (*p* = 0.40, Table 2). Blood hemoglobin (*p* = 0.17, Table 2), hematocrit (*p* = 0.13, Table 2), serum sodium (*p* = 0.23, Table 2), and plasma osmolality (*p* = 0.37, Table 2) were all in normal ranges with no significant differences in response to the diet. The HF + HS condition increased serum VLDL (Table 2, *p* = 0.01) and triglycerides (Table 2, *p* = 0.02). No significant differences were observed when assessing changes in glucose, HDL, or LDL (Table 2, *p* > 0.05). Serum creatinine in both RF + HS and HF + HS conditions was not significantly different compared to the RF + RS condition (Table 2, *p* = 0.07). Serum uric acid decreased during both RF + HS and HF + HS conditions compared to RF + RS (Table 2, *p* < 0.01).

**TABLE 3:** Physical activity monitoring after each intervention.

	RF + RS	RF + HS	HF + HS	*p*
Sedentary, min/day	1067.9 ± 85.7	1034.0 ± 103.8	1065.9 ± 103.3	0.36
Light PA, min/day	779.3 ± 119.6	789.4 ± 164.3	756.3 ± 116.0	0.80
Moderate PA, min/day	62.4 ± 19.8	59.1 ± 16.9	60.1 ± 18.3	0.98
Vigorous PA, min/day	7.7 ± 7.7	6.3 ± 7.3	6.2 ± 7.2	0.78
Very vigorous, min/day	1.6 ± 2.1	2.11 ± 4.4	2.4 ± 4.6	0.45
Step count, steps/day	6951.2 ± 2688.9	6724.1 ± 2650.5	6741.4 ± 2591.3	0.92

*Note*: Recommended fructose plus recommended salt (RF + RS), recommended fructose plus high salt (RF + HS), and high fructose plus high salt (HF + HS). *N* = 27. Data are expressed as mean ± SD.

Abbreviation: PA, physical activity.

## 4 DISCUSSION

The primary findings of this double‐blind randomized crossover trial within young healthy normotensive adults are that increased fructose consumption, when combined with high dietary sodium, results in a modest decline in urinary sodium excretion, an increase in mean arterial BP, and an increase in IL6. This suggests that increased fructose intake can make normally sodium‐resistant adults more sensitive to the BP‐raising effects of sodium. Notably, these changes were observed with 1 week of sodium and fructose loading, suggesting that habitual or longer‐term consumption of high sodium and fructose could have chronic deleterious effects on BP.

Alterations in renal sodium handling, whether arising from primary renal pathologies or disruptions in hormonal and neural control mechanisms, contribute to several hypertensive phenotypes (Textor, 2017). These changes often involve a diminished capacity of the kidneys to effectively excrete sodium, thereby leading to an expansion of extracellular volume (ECV) and fostering the onset of hypertension. Our hypothesis stemmed from prior preclinical investigations suggesting that dietary fructose could induce shifts in renal sodium handling and BP (Cabral et al., 2014; Gordish et al., 2017). Indeed, past studies have elucidated that fructose augments the expression of renal sodium transporters, thereby promoting heightened sodium reabsorption and diminished excretion (Queiroz‐Leite et al., 2012). Our study examines this mechanism in humans by assessing changes in sodium excretion across three distinct dietary conditions. Our finding that increased fructose intake can make normally sodium‐resistant adults more sensitive to the BP‐raising effects of sodium is consistent with the literature in Sprague Dawley rats where the combination of a high sodium and fructose diet led to a salt‐sensitive BP response in an otherwise salt‐resistant strain of rat (Cabral et al., 2014; Gordish et al., 2017).

To stimulate an effective dietary perturbation on the described renal and BP measures, we utilized sodium capsules and fructose‐sweetened beverages over three 7‐day periods. As expected, sodium excretion was significantly increased during the two high‐sodium conditions. Consistent with our hypothesis and existing preclinical research, adding fructose reduced sodium excretion in healthy adults. We also observed sodium‐ and fructose‐induced osmolar and free water clearance alterations. Osmolar clearance increased in the high‐sodium conditions, while adding fructose resulted in a modest decline. Salt loading induces water conservation, and our findings are consistent with previous studies that demonstrated a significant decrease in the rate of free water clearance with salt loading (Rakova et al., 2017). Adding fructose resulted in an attenuated decrease, suggesting fructose does not promote water conservation. Increased salt intake in hypertensive patients has been shown to increase glomerular filtration rate (Rossitto et al., 2021). Studies on young, healthy adults have shown that short‐term dietary salt loading can increase kidney injury markers as well as increase creatinine clearance (Barnett et al., 2022). However, our study found no significant differences in creatinine clearance, and we did not assess kidney injury markers.

One underlying mechanism implicated in the development of the SSBP phenotype in rodents when exposed to a diet high in fructose and sodium is the marked elevation in sodium hydrogen exchanger 3 (NHE3) expression (Queiroz‐Leite et al., 2012). NHE3 is an important mediator of sodium transport in the proximal tubules. This increased expression leads to an enhanced reabsorption of sodium. Dietary fructose has been shown to increase NHE3, thereby potentiating sodium transport mechanisms (Singh et al., 2008). Evidence suggests that the increase in BP is mediated by protein kinase C within the proximal tubules—a pivotal regulator of NHE3 (Yang et al., 2020). Furthermore, there is evidence indicating that excess dietary fructose can counteract the inhibitory effect of sodium on the renin‐angiotensin‐aldosterone system (RAAS) (Cabral et al., 2014; Gonzalez‐Vicente et al., 2017; Yang et al., 2020). This is corroborated by studies demonstrating that higher consumption of fructose leads to increased sensitivity of the proximal tubule to lower concentrations of Angiotensin II (AngII), resulting in greater stimulation of the RAAS (Cabral et al., 2014).

Systemic inflammation can also contribute to elevated BP. There is evidence that the SSBP phenotype is associated with an increased level of immune cell infiltration into the kidneys, which can lead to renal inflammation (Mattson, 2014). The inhibition of IL6 has been shown to reduce SSBP, and increased IL6 levels are strongly associated with increased immune cell infiltration into the kidney. This suggests that increased immune cell infiltration may impact sodium reabsorption in the kidney (Hashmat et al., 2016; Singbartl et al., 2019). Moreover, heightened renal inflammation may influence renal sympathetic nerve activity, hence contributing to the phenotype identified in our investigation. Rodent studies indicate that renal denervation markedly decreases immune cell infiltration in the kidney and blunts the hypertensive response (Xiao et al., 2015). These mechanisms may explain the effects of fructose on renal sodium handling demonstrated in this study, although this requires validation in future studies.

We assessed the impact of high fructose and salt intake on uric acid levels, lipid profiles, and BP in normotensive adults. We expected an increase in BP in the HF + HS condition since studies conducted in salt‐resistant rodents observed significant increases in BP after rodents consumed an increased fructose and sodium diet (Cabral et al., 2014). Within this cohort, we observed a modest but statistically significant increase in mean BP during the HF + HS condition. The increase in BP during the HF + HS condition may result from an impairment in urinary sodium excretion, as noted above, which would lead to an increase in ECV and therefore BP. As mentioned, it is notable that these changes were observed with a 1‐week intervention. We would anticipate a more pronounced effect with a longer intervention period.

While it is well‐established that a HS diet increases BP, which can potentially impair kidney function and raise uric acid levels (Sanchez‐Lozada et al., 2020; Sandler et al., 1989) and a HF diet is associated with elevated uric acid and BP (Zhang et al., 2020), our findings demonstrated a decrease in uric acid levels with HS or combined HS and HF intake, independent of changes in BP. This finding is consistent with short‐term studies examining different sodium intake levels and uric acid (Juraschek et al., 2016; Wang et al., 2018). We believe that combining HS and HF intake could exacerbate hyperuricemia through different mechanisms, warranting further research. The combination of HS and HF increased VLDL and triglycerides by 50% and 56%, respectively. This may be due to HS‐induced leptin resistance through the stimulation of endogenous fructose production via the aldose reductase (polyol) pathway, contributing to dyslipidemia by altering lipid metabolism (Lanaspa et al., 2018; Sanchez‐Lozada et al., 2019). Additionally, HS‐induced fluid retention may exacerbate insulin resistance, further influencing lipid metabolism (Rocchini et al., 1989). Additional studies are needed to understand these responses fully.

While this study was not statistically powered to investigate sex differences, we performed an exploratory analysis since prior research has suggested that salt‐sensitive blood pressure is more pronounced in females compared to males (Barris et al., 2023; Brinson et al., 2014; Faulkner & Belin de Chantemèle, 2020). While men had higher BPs in all three trials, there was no sex‐by‐diet interaction for mean arterial BP. Additionally, we explored how this intervention influenced nocturnal SBP dipping since an impaired nighttime dip in SBP is associated with cardiovascular disease and mortality (Yano & Kario, 2012). A reduction in SBP during sleep compared to wake periods typically exceeds 10% in young healthy adults. Previously, our lab has provided evidence that demonstrates increased dietary sodium does not impair nocturnal dipping of BP (Brian et al., 2017). In this cohort, the high salt alone condition did not have a significant effect compared to the recommended condition, consistent with our earlier findings. While we did not observe significant differences when comparing either the sodium and/or fructose loading conditions to the recommended diet, we did observe less nocturnal BP dipping (i.e., less of a reduction in BP during sleep) due to the combined increase of sodium and fructose compared to sodium loading alone. It appears BP dipping may be sex specific. In males, the dietary combination of increased fructose and sodium decreased nocturnal BP dipping compared to sodium loading alone, whereas females did not exhibit this effect, highlighting a potential sex‐specific difference. These findings suggest additional work is needed to elucidate the mechanism by which sodium and fructose impact BP dipping.

While the doses of both sodium and fructose provided in this study are above the average intake observed in the Western diet (approximately 3400 mg sodium per day and 55 g fructose per day), the upper quintile of the population consumes upwards of 5000 mg of sodium and 75 g of daily fructose intake (Mente et al., 2021; Vos et al., 2008). In one study, they reported adults consuming approximately 110 g of fructose or added sugars in their diet (Duffey & Popkin, 2008). Furthermore, consistent with previous studies in the literature and from previous studies from our lab, we find that doubling the average sodium intake has no effects on BP in the salt‐resistant population (Matthews et al., 2015). While these doses are high, we find that they are clinically relevant. In this study, we aimed to expand the current rodent literature investigating SSBP with the addition of fructose.

This study has several limitations. This study aimed to observe the effects of a combined high fructose and high salt diet, compared to high salt alone and recommended levels of salt and fructose. Some studies demonstrate that fructose alone can induce changes in BP; however, these observations are typically recorded after 6 weeks of chronic consumption (Béghin et al., 2021; Chen et al., 2010; Janssen et al., 2022; Senador et al., 2012; Vázquez‐Durán et al., 2016). We considered participant burden when designing the study and developing the a priori hypothesis. We did not control for the menstrual cycle or contraceptive usage in these premenopausal women. While this may increase variability among women, we believe this increases external validity, making the results of this study more generalizable to women across the menstrual cycle and with or without contraceptive usage. Importantly, according to the findings from rodent models, males and non‐cycle matched females do not differ considerably in terms of trait variability (Dayton et al., 2016).

## 5 SUMMARY AND CONCLUSIONS

In this randomized crossover, double‐blinded controlled trial, our goal was to determine if dietary fructose and sodium increase BP in a group of normotensive adults. Our findings have clinical and practical implications that increased dietary fructose, in conjunction with high sodium intake, decreases sodium excretion, modestly increases BP, and increases IL6. Overall, this study demonstrates that within a single week, high sodium and fructose can alter renal sodium handling, BP, and a marker of inflammation in healthy adults. Future studies are encouraged to explore how the combination of high fructose and high salt impacts other factors influencing BP regulation in humans, such as autonomic regulation and vascular stiffness.

### 5.1 Perspectives and significance

This study translated findings performed in salt‐resistant rodent models to humans. While these preclinical studies provided mechanistic insight, it was unclear if the findings applied to healthy adults. The standard Western diet is characterized by an excessive intake of sodium and fructose, both of which have been linked to the development of cardiovascular disease. However, the specific clinical implications of this combination have not yet been thoroughly investigated in humans. The findings herein highlight how sodium and fructose in the diet can influence BP through changes in renal sodium absorption in humans.

## AUTHOR CONTRIBUTIONS

R.K.M., W.B.F., and J.M.S. conceived and designed research. R.K.M., J.M.S., and N.T.R. performed experiments and analyzed data. R.K.M, J.M.S., N.T.R., M.M.W., S.C.C., and W.B.F. interpreted results of experiments. R.K.M. prepared figures and drafted manuscript. R.K.M., J.M.S., N.T.R., and W.B.F. edited and revised manuscript. R.K.M., J.M.S., N.T.R., S.C.C., M.M.W., and W.B.F. approved final version of manuscript.

## FUNDING INFORMATION

This work was supported in part by the American Physiological Society Porter Fellowship. This publication was also made possible by the Delaware COBRE in Cardiovascular Health.

## CONFLICT OF INTEREST STATEMENT

No conflicts of interest, financial, or otherwise, are declared by the authors.

## ETHICS STATEMENT

Study protocols and procedures were approved by the Institutional Review Board of the University of Delaware (IRB#1617405) and conformed to the provisions of the Declaration of Helsinki.

## Data Availability

The data that support the findings of this study are available from the corresponding author upon reasonable request.
